# Optically read Coriolis vibratory gyroscope based on a silicon tuning fork

**DOI:** 10.1038/s41378-019-0087-9

**Published:** 2019-10-21

**Authors:** N. V. Lavrik, P. G. Datskos

**Affiliations:** 1 Center for Nanophase Materials Sciences, Oak Ridge National Laboratory, Oak Ridge, TN 37931-6054 USA; 2 Mechanical and Thermal Engineering Sciences, National Renewable Energy Laboratory, Golden, CO 80401 USA

**Keywords:** Nanoscience and technology, Nanoscale devices, Engineering

## Abstract

In this work, we describe the design, fabrication, and characterization of purely mechanical miniature resonating structures that exhibit gyroscopic performance comparable to that of more complex microelectromechanical systems. Compared to previous implementations of Coriolis vibratory gyroscopes, the present approach has the key advantage of using excitation and probing that do not require any on-chip electronics or electrical contacts near the resonating structure. More specifically, our design relies on differential optical readout, each channel of which is similar to the “optical lever” readout used in atomic force microscopy. The piezoelectrically actuated stage provides highly efficient excitation of millimeter-scale tuning fork structures that were fabricated using widely available high-throughput wafer-level silicon processing. In our experiments, reproducible responses to rotational rates as low as 1.8 × 10^3^° h^−1^ were demonstrated using a benchtop prototype without any additional processing of the raw signal. The noise-equivalent rate, *Ω*_NER_, derived from the Allan deviation plot, was found to be <0.5° h^−1^ for a time of 10^3^ s. Despite the relatively low *Q* factors (<10^4^) of the tuning fork structures operating under ambient pressure and temperature conditions, the measured performance was not limited by thermomechanical noise. In fact, the performance demonstrated in this proof-of-principle study is approximately four orders of magnitude away from the fundamental limit.

## Micro-scale Coriolis gyroscope: all-mechanical

A Coriolis force vibratory gyroscope that relies entirely on mechanics demonstrates reduced noise. Micro-scale gyroscopes are used in a wide range of devices, and typically rely on mechanical and electrical principles to operate. However, the electrical component of their operation can interfere with the device, causing noise. Now, N.V. Lavrik from Oak Ridge National Laboratory and P.G. Datskos from National Renewable Energy Laboratory demonstrate an all-mechanical device with reduced noise, without the need for signal processing. Key to their design is the use of an optical readout, and a design that takes inspiration from how insects navigate. They achieve a reproducible response to rotational rates as low as 1.8 × 10^3^° h^−1^, and a noise equivalent rate of 0.6° h^−1^ for a time of 10^3^ s.

## Introduction

Over the past two decades, micromechanical inertial sensing devices, such as accelerometers and gyroscopes, have been the subjects of extensive research^1–3^. Although accelerometers and gyroscopes based on microelectromechanical systems (MEMS) have already been implemented in many commercial devices, there is still both fundamental and industrial interest in continued research efforts in this area. This interest is stimulated primarily by the need for miniature inertial sensing devices with figures of merit orders of magnitude better than those of the existing state-of-the-art. Miniaturizing gyroscopes using innovative designs and microfabrication processes is an attractive solution to inertial sensing that opens up new market opportunities^2^. Further advances in this area are beneficial for not only navigation applications but also fundamental geophysical studies and even tests of general relativity theory and laws of gravity and inertia.

Micromachined accelerometers and gyroscopes are some of the most important types of silicon-based devices successfully commercialized after the establishment of complementary metal oxide-semiconductor technology^1^. Since the first demonstration of a micromachined gyroscope^4^, similar devices have been implemented using surface, bulk, and hybrid surface-bulk micromachining approaches. Although numerous gyroscope designs based on a variety of physical principles have been described, the majority of MEMS gyroscopes are based on vibrating mechanical elements that successfully utilize the effect of the Coriolis force. These devices, known as Coriolis vibratory gyroscopes (CVGs), are based on the transfer of energy between two vibration modes of a structure caused by Coriolis acceleration^5^. When rotated, a vibrating element is subjected to a sinusoidal Coriolis force that causes secondary vibration orthogonal to the original vibrating direction. By sensing the secondary vibration, the rate of rotation can be detected^6^. Interestingly, a similar principle is used by nature in certain insects that have vibrating dumbbell-shaped organs, known as halteres, to aid their flight^7,8^.

A number of MEMS-based gyroscopic devices suitable for less demanding applications are now commercially available^9^. However, low-cost and high-performance devices are not yet available. Current state-of-the-art micromechanical CVGs require a few orders of magnitude improvement in stability to be useful for navigation applications without assistance from the Global Positioning System. It is important to emphasize that the relatively poor performance of existing MEMS gyroscopes is often limited by the multiple sources of readout noise and drifts present in practical devices^10^. Although the more sophisticated MEMS gyroscope designs developed and implemented more recently have exhibited improved performance^11^, they are also associated with substantially increased fabrication and operational complexity.

The various sources of noise in micromechanical systems^12^ and nanoscale resonators^13^ have been extensively studied with regard to their impact on the performance of MEMS sensors. It is well-established that evaluation of thermomechanical noise in CVGs can be useful in assessing the ultimate limit of their performance^14^ and, in turn, making conclusions about potential improvements in practically achievable figures of merit^15^. However, the real-world performance of any MEMS gyroscope is typically affected more strongly by the multiple additional sources of noise and drifts associated with the readout and overall mechanical stability of the device rather than by thermomechanical noise. As a result, various advanced designs and transduction methods that minimize drifts and noise in MEMS have been explored to implement CVGs with improved performance^16,17^. It should be noted that these strategies focus mostly on complex designs that increase the *Q* factor, optimize the transduction efficiency, improve the sensitivity of the electronic readout method, and facilitate integration of CVGs onto MEMS chips. On the other hand, the important question of whether miniature CVGs with navigation-grade performance are fundamentally feasible has received relatively little attention in the recent literature.

In our present proof-of-principle study, we use a differential optical readout and focus on a micromechanical CVG design that closely mimics the halteres, the gyroscopic sensory organs of insects^7,18^. This type of design, with its inherent simplicity, can be straightforwardly implemented using well-established wafer-level processing while also addressing traditional challenges in sensing extremely small mechanical deformations. More importantly, due to advances in additive manufacturing, similar resonating structures can now be printed^19^ in three dimensions using titanium-based alloys^20^. Furthermore, recent advances in coherent light sources point to a promising path toward optical readout of CVGs with measurement of mechanical displacement that is below the shot-noise limit and that has a substantially improved signal-to-noise ratio^21^.

## Results

### Analytical models of CVGs

**Fig. 1:**
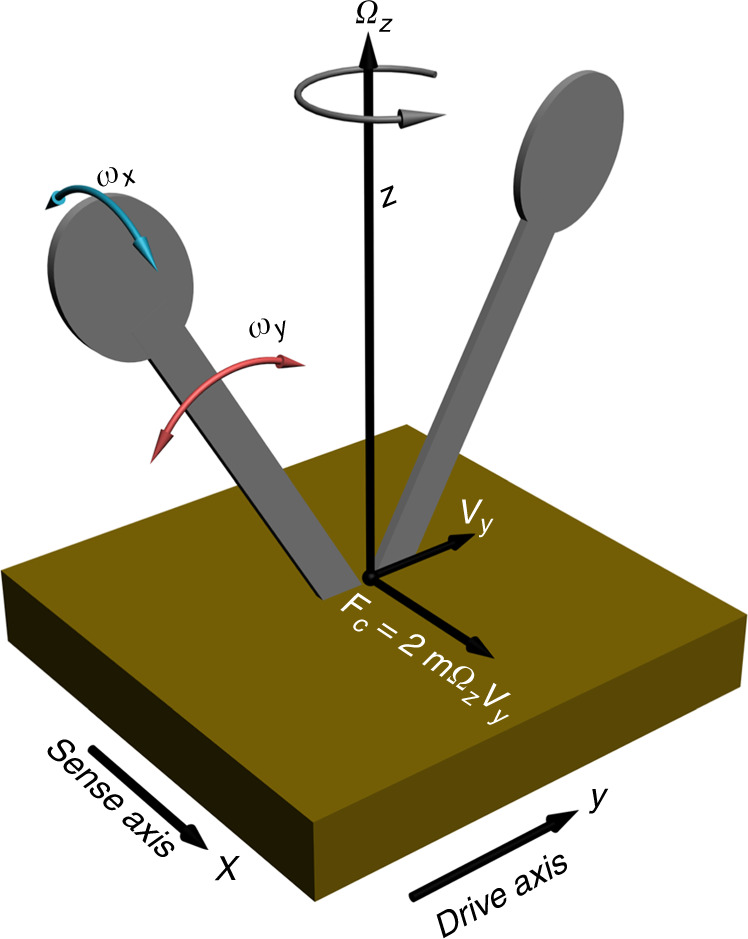
Physical model of a double-tine CVG. The CVG is on a platform that rotates horizontally, and the tines are actuated perpendicularly to the platform. Note that this model is applicable to a wide variety of resonator shapes, such as a tuning fork or similar

Typically, the operation of a CVG is described by a simplified model that involves a pair of coupled resonating beams (tines) that can oscillate in two mutually orthogonal planes (Fig. 1). The Coriolis force can be detected as the differential deflection of the tines in the direction perpendicular to the elliptical structures shown in Fig. 1. The dynamics of oscillating structures in the CVG studied in this work can be described by two coupled second-order differential equations^22^. The equations of motion for the combined system shown in Fig. 1 under the influence of a periodic external force, *F*_*y*_, and an angular frequency, *Ω*_*z*_, have been described previously^22^.

An applied periodic force, *F*_*y*_, causes the tines to oscillate along the drive axis with a maximum amplitude, Δ*y*, and a maximum velocity, *v*_*y*_ = *ω*_*y*_Δ*y*. When a rotation rate, *Ω*_*z*_, is introduced along the *z*-axis, the Coriolis force, *F*_c_ = 2 *mΩ*_*z*_*v*_*y*_, forces the tines to oscillate along the sense axis with an amplitude, Δ*x*, related to the rotation rate, *Ω*_*z*_, by ref. ^23^

1 $${\mathrm{\Delta }}x = \frac{{2\,{\mathrm{\Delta y}}\,\omega _y}}{{\omega _x^2\sqrt {\left( {1 - \frac{{\omega _y^2}}{{\omega _x^2}}} \right)^2 + \frac{1}{{Q_x^2}}\frac{{\omega _y^2}}{{\omega _x^2}}} }}{\mathrm{\Omega }}_z$$

where *Ω*_*z*_ is the angular frequency along the *z*-axis, *ω*_*y*_ = (*k*_y_/*m*)^1/2^ and *ω*_*x*_ = (*k*_*x*_/*m*)^1/2^ are, respectively, the resonance frequencies of the tines along the *y* axis (drive axis) and *x* axis (sense axis), *Q*_*y*_ and *Q*_*x*_ are, respectively, the quality factors of the oscillation along the drive axis and sense axis, and *m* is the effective mass of the oscillator.

To determine the sensitivity of the CVG studied in this work and its ultimate performance limit, we need to evaluate the noise-equivalent rotational rate, *Ω*_NER_, which is defined as the rotational rate at which the signal-to-noise ratio of the CVG equals unity^24^. Using Nyquist’s relation^14,23^ for the spectral density of the fluctuating force related to the CVG, we obtain for a bandwidth, *B*, the thermal noise-equivalent rate, *Ω*_NER_^23^.

2 $${\mathrm{\Omega }}_{\mathrm{NER}} = \sqrt {\frac{{k_{\mathrm{B}}TB\omega _x}}{{m\,{\mathrm{\Delta }}y^2\,\omega _y^2Q_x}}}$$

In inertial navigation systems, the measured rotation rate is integrated over time to determine the orientation, and depending on the integration time, different noise sources can introduce errors^25,26^. Thermomechanical noise represents the lowest noise floor fundamentally achievable in any MEMS CVG. Therefore, Eq. (2) can be used to analyze the fundamentally limited performance of a CVG as a function of resonance parameters, mass, and driving amplitude.

The tuning fork used in the present work can be excited in such a way that two equal-proof masses are forced into antiphase oscillations along the drive axis. The oscillation due to the driving force is parallel to the support plane, whereas the sensing direction (sense axis) is perpendicular to the driving force. Under these conditions, the oscillating tines respond to angular rate inputs in directions opposite to each other because of opposite Coriolis forces. The differential response is expected to be nearly immune to linear acceleration, which causes deflection of the tines in the same direction.

From Eq. (2), the fundamentally limited performance of the CVG has a straightforward dependence on the values of the proof mass, driving amplitude and quality factor of the sensing tines. From this perspective, it would be beneficial to have the largest possible tuning fork structure driven with the largest possible amplitude while also minimizing any losses that negatively impact the quality factor. However, these measures would counteract the goal of miniaturization. Therefore, there is a nontrivial trade-off between the concept of scaling down CVG designs and their optimization from the standpoint of fundamentally limited performance. This trade-off, along with the multidimensional nature of the parameter space involved in Eq. (2), represents the main challenge in optimizing CVG designs. Furthermore, all the parameters in Eq. (2), particularly the proof mass, resonance frequencies, and driving amplitude, are interrelated and impose boundaries on the values for realistic designs.

To address this challenge, we made two important assumptions regarding the CVG resonance frequencies, *ω*_*x*_ and *ω*_*y*_. First, we assumed that the difference Δ*ω* between *ω*_*x*_ and *ω*_*y*_ is relatively small. This assumption implies mode-matching resonance conditions for the drive and sense modes, which is known to provide maximum gain and sensitivity due to a *Q*-amplified sense response^27^. However, in mode-matched operation, some noise and drift sources tend to also be amplified. The sense mode being shifted from the drive mode leads to improved thermal stability while also reducing gain and sensitivity. Therefore, a small difference between *ω*_*x*_ and *ω*_*y*_ can be considered optimal. Second, we assumed that there is a limited range of practically feasible resonance frequencies for miniature CVGs. While this range is difficult to rigorously determine, it can be estimated from previous experimental and theoretical studies on cantilever-like structures and MEMS devices. Most CVGs operate in the kHz range^28–30^ due to the susceptibility of lower-frequency mechanical resonators to external vibrations. Microcantilevers used in scanning probe microscopy have resonance frequencies in the range from ~2 to 200 kHz. The lower end of this range is of particular interest to us because the lower the frequency is, the greater the amplitude that can be achieved in a CVG for a given *Q* factor, proof mass, and driving force.

**Fig. 2:**
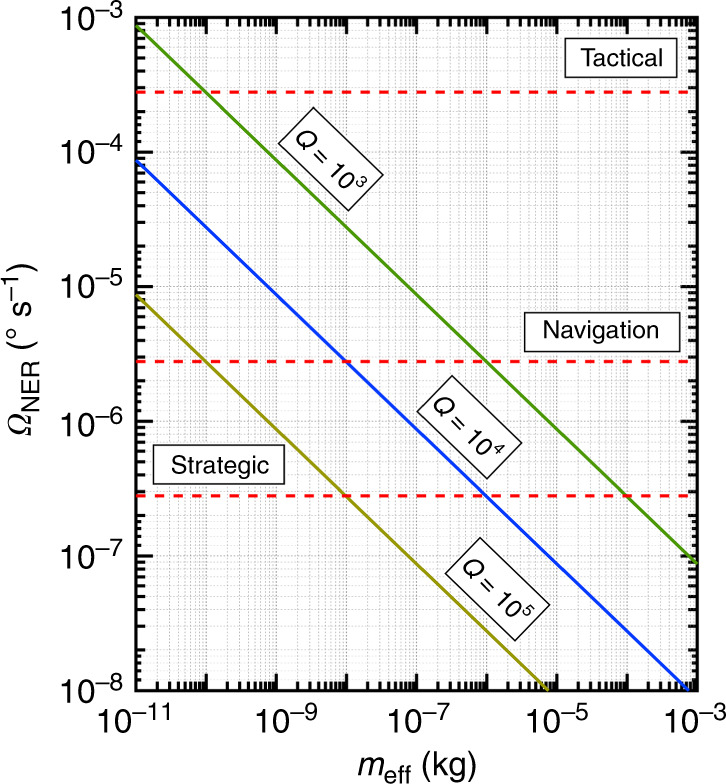
Calculated NE*Ω* as a function of CVG proof mass, *m*, for different quality factors, *Q*. The driving amplitude is 10^–3^ m, and the drive and sense-mode frequencies are *ω*_*y*_ = 2.1 × 10^4^ rad s^−1^ and *ω*_*x*_ = 1.7 × 10^4^ rad s^−1^, respectively. The dashed lines show the NEΩ for different grade gyroscopes

To guide our designs, we calculated the noise-equivalent rotation (NER) limited by the thermomechanical noise as a function of proof mass for CVGs with different *Q* factors while maintaining the resonance frequency and driving amplitude constant. In Fig. 2, we show the calculated *Ω*_NER_ for a CVG as a function of effective mass, *m*_eff_, and different quality factors. In these calculations, we used a realistic (for our design) actuation amplitude, Δ*y* = 10^–3^ m, drive and sense-mode frequencies, *ω*_*y*_ = 2.1 × 10^4^ rad s^−1^ and *ω*_*x*_ = 1.7 × 10^4^ rad s^−1^. In Fig. 2, we also show the limits of performance required for gyroscopes for different applications (e.g., consumer, automotive, tactical, and navigation)^16,25,31^.

Figure 2 shows that navigation-grade CVGs are fundamentally possible when the proof mass is >10^−6^ kg, even with *Q* factors in the range from 10^3^ to 10^5^. It should be noted that the dependence of *Ω*_NER_ on the proof mass shown in Fig. 2 is likely to overestimate the performance of smaller CVGs because their excitation with millimeter-scale amplitudes is not necessarily feasible.

**Fig. 3:**
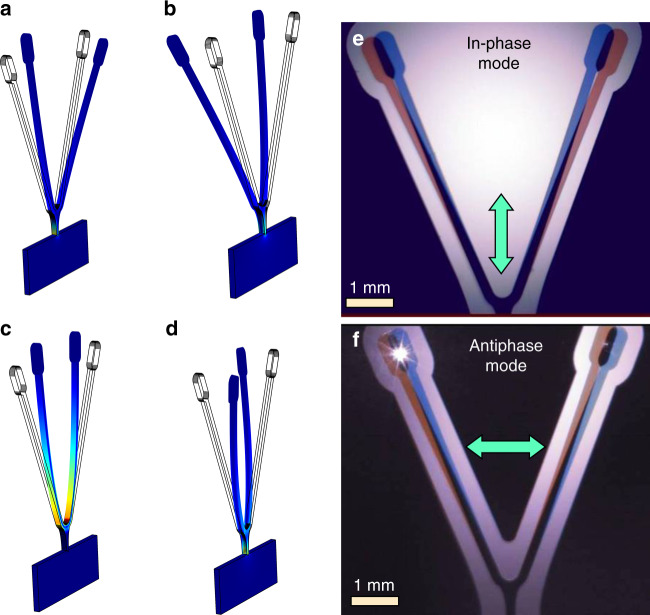
Eigenfrequency analysis for the CVG structures showing the four lowest modes. **a** in-phase (bending) drive mode of 2.01 kHz, **b** in-phase (bending) sense mode of 2.64 kHz, **c** antiphase (bending) drive mode of 3.2 kHz, and **d** antiphase (torsional) sense mode at 3.38 kHz. Also shown is the stroboscopic visualization of the driving modes: **e** in-phase motion of the tines (asymmetric mode) and **f** out-of-phase motion of the tines (symmetric mode). The double arrow indicates the direction of the applied excitation

A series of iterative parametric sweeps performed in COMSOL allowed us to identify a CVG geometry with a proof mass on the order of 10^−6^ kg and resonance frequencies close to 3 kHz (Fig. 3). The total mass of a silicon tuning fork with this geometry was calculated to be 2.45 × 10^−6^ kg. It is important to note that effective proof masses and bending rigidities are somewhat different for each mode. As a reasonable approximation, we can calculate the effective mass for antiphase modes by assuming that each tine is analogous to a cantilever with an effective mass equal to one-fourth of its total mass. For the silicon fork shown in Fig. 3, this approximation gives an effective proof mass of ~4.2 × 10^−7^ kg. The antiphase drive and sense-mode spring constants are calculated to be 170 and 190 N m^−1^, respectively.

Our eigenfrequency analysis performed in COMSOL revealed the four lowest modes that are of the greatest importance to the operation of the designed CVG. In accordance with the type of deformation involved in these four modes (shown in Fig. 3a–d), we will refer to them as (a) in-phase drive, (b) in-phase sense, (c) antiphase drive, and (d) antiphase sense modes. The values of the resonance frequencies corresponding to these modes are (a) 2.01 × 10^3^ Hz, (b) 2.64 × 10^3^ Hz, (c) 3.2 × 10^3^ Hz, and (d) 3.38 × 10^3^ Hz.

It should be noted that the exact shape of the deformations associated with each mode is very important for two main reasons. First, analysis of the deformations allowed us to differentiate in-phase and antiphase modes and conclude that the Coriolis response should contribute to the antiphase sense mode when the antiphase drive mode is excited. Second, we can identify the regions where the greatest deformations and, in turn, stresses (color coded red corresponding to the maximum stress in Fig. 3a–d) are localized. The latter is key to further optimization of the CVG designs in terms of mode coupling, mechanical robustness, and ability to operate with very large vibration amplitudes. While such optimization is beyond the scope of the current study, our simulations indicate that among the four modes, the in-phase drive and sense modes are associated with the most significant stresses localized in the neck region of the tuning fork. Because the in-phase drive and sense modes can be readily excited by linear acceleration, an increase in the cross-section of this region and/or a decrease in its length can lead to CVG designs that are more immune to shock and linear acceleration. At the same time, a narrower neck region would lead to reduced clamping losses and, in turn, higher *Q* factors. Finally, frequency spreads between various modes are strongly affected by the exact shape and sizes of the neck region. In the tuning fork design selected for fabrication, the antiphase drive and sense modes are separated by only 180 Hz according to our simulations. This frequency spread can be further decreased by making the cross-section of the neck region somewhat smaller. However, the effect of a narrower neck is not limited to a reduced frequency spread between these modes. It will also lower the frequencies of in-phase sense and drive modes and make the whole structure more susceptible to linear acceleration and mechanical shocks. Therefore, further improvements in the CVG design would involve a nontrivial optimization of the neck size and overall shape geometry of the structure.

### Experimental characterization

**Fig. 4:**
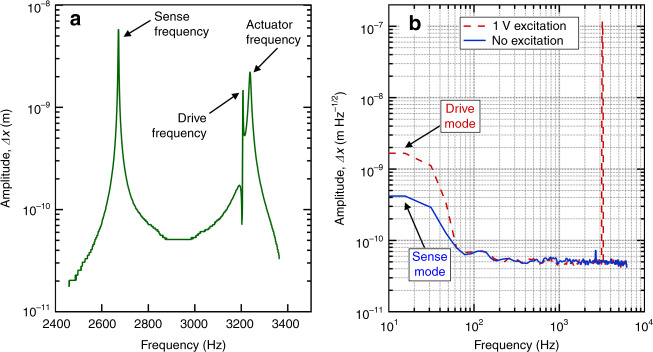
CVG response as a function of frequency. **a** Frequency response of the actuated CVG. The sense-mode frequency was smaller than the drive-mode frequency, which, in turn, was smaller than the piezo-actuator resonance: *f*_sens_ < *f*_dr_ < *f*_piezo_. **b** Frequency response of the CVG with and without excitation demonstrating the readout noise, driving and sensing modes, and coupling between modes during excitation. The readout noise is <10^−10^ m Hz^−1/2^. The actuation does not affect the baseline noise for *f* > 100 Hz. Mode coupling can cause the sensing tine to oscillate when CVG is actuated

We verified that the implemented CVG structures could be resonantly driven with amplitudes approaching 10^−3^ m near a frequency of 3 kHz. Depending on the way that the tuning fork was mounted in the piezo-driven stage, asymmetric (in-phase) or symmetric (antiphase) drive modes could be excited. In Fig. 3e, f, we also show optical images of the tuning fork oscillating in the asymmetric mode (Fig. 3e) and symmetric mode (Fig. 3f) under stroboscopic lighting. Owing to stroboscopic lighting, the tines captured in each image in Fig. 3e, f correspond to different phases of the oscillation. The approximate amplitude of the drive-mode oscillation was estimated from these images to approach 10^−3^ m. An overview of the frequency response of the actuated CVG is shown in Fig. 4a. The frequency response was measured using a lock-in amplifier with a sweep generator connected to the position-sensitive detector (PSD) and the piezo-actuator.

The measured frequency spectrum shows a clear sign of resonance response at ~2.67 kHz. Taking into account our COMSOL simulations, we can conclude that this resonance corresponds to the in-phase sense mode. From the measured frequency response shown in Fig. 4a, we determined the value of *Q*_s_ = 6.8 × 10^3^. On the other hand, the antiphase sense mode corresponds to dynamic deformations under Coriolis force.

The resonance feature shown in Fig. 4a at 3.207 × 10^3^ Hz matches the frequency used to excite the antiphase drive mode in our previous experiments (Fig. 3f) and is close to the prediction for the antiphase drive mode from our eigenfrequency analysis. We want to point out that deflection of the tines in the drive mode does not directly contribute to deflection of the probing beam, which can explain the absence of the conventional Lorentzian shape in the frequency response around the drive mode. Finally, the more distinct resonance at 3.238 × 10^3^ Hz was due to the resonance in the sample stage coupled to the piezo-stack.

In Fig. 4b, we show a comparison of frequency responses measured for the CVG with and without excitation in a wider range. As shown in Fig. 4b, the optical readout has a noise floor below 10^−10^ m Hz^−1/2^ in the frequency range above 100 Hz. In this frequency range, there was no measurable increase in the noise floor when the drive mode was excited. Nonetheless, excitation of the drive mode caused a significant increase in the noise in the low-frequency part of the spectrum (below 100 Hz). This increase can be explained by substantial mode coupling in the implemented device.

In addition, we characterized the CVG by measuring the temporal responses to rotation using both nondifferential and differential optical readouts. The motion of each sensing tine was detected either separately using a nondifferential readout or as the difference between Coriolis responses from the two tines using a differential readout scheme.

**Fig. 5:**
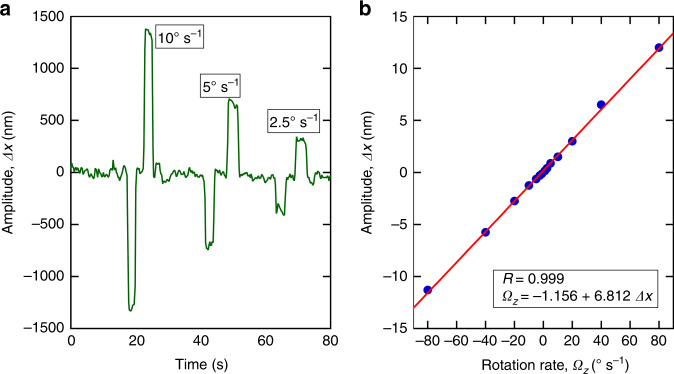
Temporal and rotational response of the CVG. **a** Responses of the CVG measured using nondifferential measurements. **b** A typical calibration curve obtained for a CVG structure in a nondifferential regime. The optical readout gain is 2.5 mV nm^−1^

The CVG was driven at 3.207 × 10^3^ Hz by applying an excitation voltage of 1 V to the piezo-stack and exciting the symmetric (antiphase) mode. This mode has the advantage of generating the Coriolis forces exerted on each tine in the opposite direction, while any spurious linear acceleration generates forces in the same direction. Figure 5a shows the temporal responses obtained in the nondifferential readout over a period of 80 s of continuous measurement by applying rotation rates *Ω*_*z*_ of 2.5, 5, and 10° s^−1^.

A typical calibration plot obtained using the nondifferential readout is shown in Fig. 5b. The calibration plot can be approximated by a linear trend with a negligible offset and a correlation coefficient of 0.999.

**Fig. 6:**
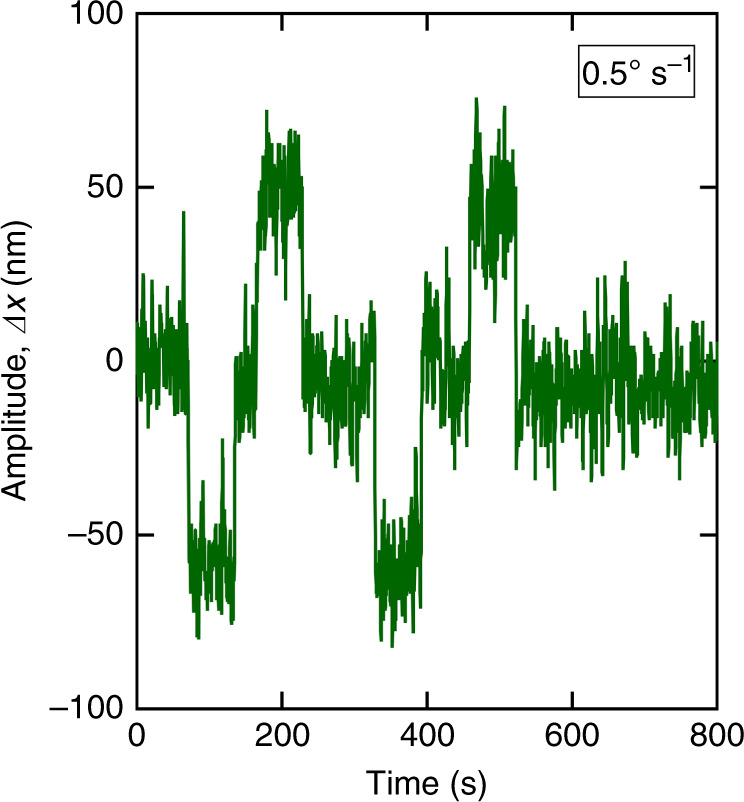
Temporal response of the CVG. Responses of the CVG measured using the differential optical readout method

From the data obtained with the nondifferential measurement method, we estimated the lowest resolvable rotational rate to be <0.5° s^−1^. Next, we recorded the responses of the CVG to rotation using the differential measurement method (Fig. 6). Compared to the nondifferential measurements, the differential measurements resulted in a signal-to-noise ratio approximately three times higher. The lowest rotational rate resolvable with differential readout was estimated to be <0.16° s^−1^. In this work, the maximum rotational rate we measured was 9 × 10^3^° s^−1^, which gives a dynamic range^32^ of ~90 dB. For our experimental conditions, using Eq. (2), we estimated the thermal noise-equivalent rate, *Ω*_NER_, to be 4.4 × 10^−7^° s^−1^ and a scale factor [Eq. (1)] of ~8.1 × 10^−9^ m (° s^−1^)^−1^.

**Fig. 7:**
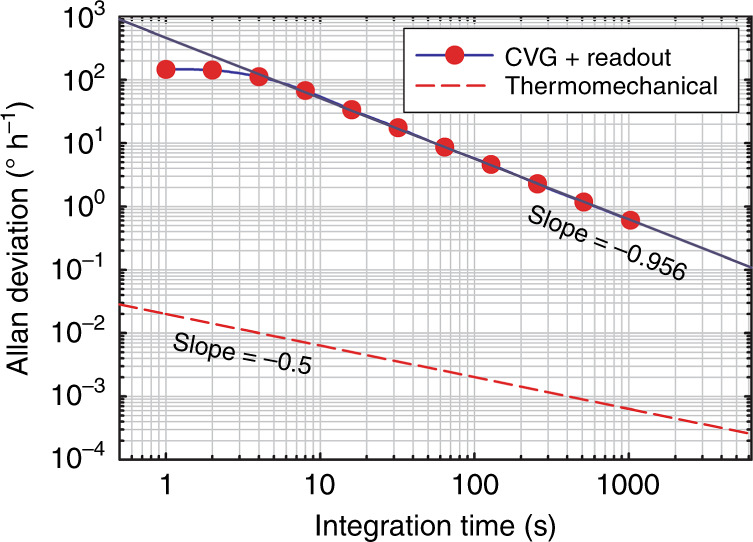
Allan deviation plot of the CVG. The response of the CVG was measured using differential measurements. The dashed curve corresponds to the estimated thermomechanical noise of the CVG

To quantify the performance of the CVG more rigorously and characterize its Allan variance, we measured the baseline for a time period of 10^3^ s. Figure 7 shows the resulting Allan deviation (solid curve) as a function of time, *τ*. The Allan deviation fits on a straight line with a slope of −0.956 for times >4 s. For shorter times, the observed leveling off of the Allan deviation is due to the lock-in amplifier integration time (0.3 s) used in our measurements. The slope of approximately −1 in the Allan deviation plot is characteristic of quantization noise^33^. Therefore, for the differential measurements, the gyroscope with our optical readout exhibits a quantization noise of 0.11° and a bias instability of <0.5° h^−1^. The dashed curve corresponds to the estimated thermomechanical noise of the CVG, with a theoretical angle random walk of 2.1 × 10^−5^° h^−1/2^.

## Discussion

In the current proof-of-principle study, we used an open control loop system to drive a piezo-transducer. When operated in a controlled laboratory environment (*T* = 22 ± 1 °C), it provided stable amplitudes of the tuning fork tines to within 0.1%. Furthermore, using stroboscopic visualization, we were able to visually monitor the tuning fork motion. However, a closed control loop will be required in the actual implementation of a fieldable gyro based on the principles described in this work. We envision that in an actual practical device, the amplitude of both the actuating and sensing tines will be read using similar optical methods. We should point out that high precision motion control using optical PSD technology has already been successfully implemented in various commercial devices, such as the latest generation of scanning galvanometers, which exhibit very high stability with a reproducibility of 10^−6^ rad^34^.

An important question arises regarding the key factors responsible for the experimentally measured noise present in our CVG prototype at levels 3–4 orders of magnitude above the thermomechanical noise limit. Depending on the averaging time, a variety of stochastic processes can affect the overall noise level and stability of a CVG. From a purely phenomenological viewpoint, these stochastic processes can be classified as angle random walk, correlated noise, bias instability, and rate random walk. For MEMS gyroscopes in general, the three most commonly discussed noise mechanisms are thermal noise, electronics flicker noise, and accumulation of white noise^22^. As applied to the optically read CVG studied in our present work, the flicker noise manifests itself in both laser noise and PSD noise. Laser noise is known to be a dominant factor that limits the spatial accuracy and stability of optical readouts at the level of tens of picometers, which is consistent with our experimentally measured frequency responses shown in Fig. 4a, b. To approach the theoretical limit of 1.6 × 10^−4^° h^−1^, surmounting the limitations imposed by laser noise would be critical.

In the present study, we used an “optical lever” readout assembled in-house using readily available off-the-shell components without any further optimization. It is well-established that the dominant noise source in unoptimized optical readouts, such as the one used in the present study, is laser noise^12^. The readout noise floor in this work was approximately < 10^−10^ Hz^−1/2^. In our previous studies, we have shown that one way to overcome the laser noise threshold is to operate laser diodes slightly below their lasing threshold^35^. In fact, optimized optical readouts can achieve very low noise floors and are comparable to other types of MEMS readouts. For example, an optical readout of 35–125 μm long cantilevers provided a defection sensitivity better than 10^−15^ m Hz^1/2^^36^. Commercially implemented optical readouts in Doppler vibrometers can reach a resolution of 30 fm Hz^−1/2^^37^. Commercially available atomic force microscopes with an optical readout routinely reach a noise level of ~10^−12^ m^38^. Perhaps a promising path toward optical readouts of CVGs with substantially improved signal-to-noise ratios^21^ is adaptation of approaches developed recently in the areas of gravitational wave detection and optical quantum computing.

Our analysis indicates that navigation-grade performance (10^−2^° h^−1^) is fundamentally feasible for a CVG with a proof mass in the 10^−6^ kg range driven to millimeter-scale amplitudes at frequencies of a few kHz. According to theoretical and computational guidance, we implemented an entirely mechanical silicon CVG structure with a thermomechanical noise floor well below navigation-grade performance. However, the experimentally determined performance of the implemented CVG was not limited by the fundamental noise source. In particular, the smallest resolvable rotational rates measured were ~0.16°s^−1^, whereas our analysis of the Allan deviation plot revealed a bias instability noise of ~0.5° h^−1^. It can be concluded that these experimentally determined figures of merit are limited by the noise and instabilities of the optical readout, which are on the order of a few nanometers in terms of detectable deflections of the CVG tines. This conclusion points to two immediate strategies for developing a miniature CVG approaching navigation-grade performance. First, larger responses can be achieved by increasing the *Q* factors of both drive and sense modes when the CVG is operated under vacuum conditions. Second, the readout noise can be minimized by using the same coherent source to generate two light beams for the two channels of the differential readout. Additional improvements can be achieved by combining signals from different coherent sources as well as by averaging signals from several tuning fork structures^32,39^. Optimization of the CVG design can further reduce the noise and increase performance. In Table 1, we highlight specifications and performance metrics for the CVG studied in this work along with alternative existing state-of-the-art gyroscope technologies.

**Table 1:** Performance metrics of the optically read micromechanical CVG and state-of-the-art gyroscopes^40,50–52^

Parameter	Optically read CVG	FOG^a^	MEMS	MEMS DRG^b^	Units
Dimensions					
Sensing element	(8 × 5 × 2.5) × 10^−11^	(8 × 6 × 7) × 10^−9^	(4 × 4 × 3) × 10^−12^	(0.03)^2^ × 5 × 10^−4^*π*	m^3^
Gyroscope	0.2 × 0.4 × 1	(2 × 7 × 7) × 10^−6^	(1 × 1 × 4) × 10^−7^	(1 × 1 × 4) × 10^−7^	
Mass					
Sensing element	2.45 × 10^−6^				kg
Gyroscope	~10 (lab system)	<1.5 × 10^−2^	5 × 10^−2^	10^−2^	
Drive mode	3.238 × 10^3^		~10^4^	~2 × 10^3^	Hz
Sense mode	2.670 × 10^3^		~10^4^	~2 × 10^3^	Hz
Minimum displacement	2.5 × 10^−9^	N/A	<10^−14^	<10^−14^	m
Quantization noise	1.1 × 10^−1^	<10^−3^	<10^−2^	<10^−3^	°
Bias instability	<5 × 10^−1^	<5 × 10^−3^	<10^−1^	0.04	° h^−1^
Angle random walk	~10	<10^−2^	<10^−1^	10^−2^	° h^−1/2^
Range	±9 × 10^3^	±10^3^	±10^3^	±10^2^	° s^−1^

^a^Fiber optic gyroscope

^b^Disc resonator gyroscope

In the present study, the CVG with an optical readout was found to exhibit a bias instability of <0.5° h^−1^, which is a factor of ~50 lower than the performance of 0.01° h^−1^ for state-of-the-art gyroscopes. For example, a MEMS disk resonator gyroscope^40^ was recently shown to have a bias instability of <0.04° h^−1^ with a long decay time of 74.9 s. This impressive performance for a MEMS gyroscope requires complex mechanical structures with a nontrivial fabrication process. The fabrication of the CVG structures we report in this work are relatively simple, and since the sensitivity of the optical readout can be substantially improved, we expect that in future studies, the performance of the CVG described here will also be improved. For example, in the present study, the position sensitivity was < 10^−10^ Hz^−1/2^, and achieving a 50× or better improvement in the performance of the CVG gyro will require an optical readout sensitivity of ~10^−12^ m Hz^−1/2^. This requirement seems realistic since optical readout sensitivities of <10^−14^ m have already been demonstrated in commercially available optical readouts^37^.

A potential path to miniaturization for the CVG based on the principle described in this work is clearly indicated by recent advances in on-chip optical processing. Optical readouts have already been made smaller (digital versatile disc and Blu-Ray players use miniaturized optical readouts), and on-chip integration of optical sources is an active area of research. In a recent work, the combination of integrated silicon photonics and MEMS was used to demonstrate position measurement on the picometer scale under ambient conditions^41^. The path toward further miniaturization of a device based on the MEMS CVGs described in this work is directly linked to continued advances in on-chip optical processing. While fully integrated photonic circuits are not a mature technology yet, a number of complex on-chip optical systems have already been demonstrated^42–46^. Ultimately, this work has the potential to make feasible optical readouts that integrate multiple lasers sources, optical elements and optical sensing and electronic processing on a chip with a footprint similar to currently commercialized MEMS gyroscopes.

Finally, in the present proof-of-principle study, we used a relatively simple design and fabrication process of purely mechanical resonating structures that can be used as gyroscopes with an optical readout. Although similar designs have been studied before, the combination of a MEMS CVG with an optical readout offers new opportunities in both research and applications. As photonic circuits continue to improve and be integrated on-chip with mechanical devices, success on the path toward CVG miniaturization will strongly depend on the implementation of suitable materials, photon sources, optical sensing systems, and processes compatible with foundry-level fabrication^47^.

## Materials and methods

To overcome the limitations and drawbacks of previous CVG designs, we focused on an approach that takes advantage of silicon resonating structures that can be batch-fabricated using relaxed design rules due to the absence of on-chip electronics. Using well-established photolithographic patterning and wafer-scale processing^48^, we implemented resonator geometries compatible with large oscillation amplitudes and, in turn, oscillation velocities. Furthermore, a modular optical readout based on discrete components eliminates potential electronic interference without increased fabrication cost and complexity.

### CVG design and finite-element analysis

To generate tuning fork geometries for fabrication, we used the following rationale. We focused on a symmetric geometry with an anchoring region crossing the symmetry plane to minimize clamping losses using an analogy to a classic macroscopic tuning fork. A short neck region with a curved geometry provided mechanical coupling between the two tines. Smooth transitions between different regions of the CVG structure were aimed at minimizing localized stresses and reducing the likelihood of mechanical failures of the CVG when driven to high amplitudes or subjected to mechanical shocks. The nearly square cross-section of the tines ensures that their flexural rigidities in two mutually orthogonal planes corresponding to the drive and sense modes are approximately the same. Because both the effective proof masses and bending rigidities corresponding to the drive and modes are similar, these modes have similar frequencies and can be made to match.

The vibrational modes corresponding resonance frequencies inherent to a specific tuning fork geometry were quantified by performing eigenfrequency analysis in COMSOL multiphysics software. Our models relied on linear elastic approximations. To calculate resonance frequencies as a function of the tuning fork size and shape, we compiled a series of three-dimensional models and carried out geometrical sweeps.

### Microfabrication

**Fig. 8:**
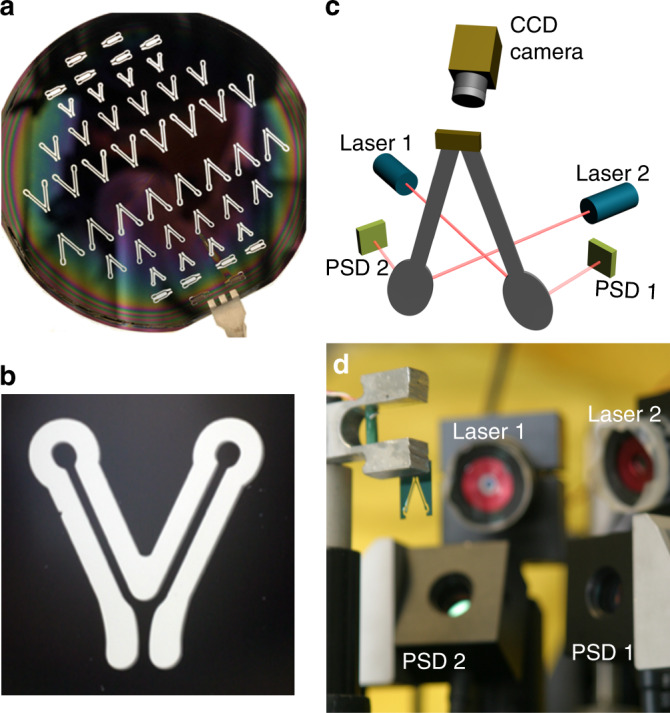
Microfabricated CVG and experimetal setup. **a** Si wafer with the microfabricated CVG structures. For CVG fabrication, we used 100-mm-diameter Si wafers that were 250-μm thick. **b** A close-up of an individual CVG structure. **c** Schematic illustration of the experimental setup, and **d** a photograph of the implemented differential “optical lever” readout. The optical setup was mounted on a precision turntable for rotational rate measurements. The piezo-driven holder vibrates vertically at its point of attachment to the silicon tuning fork. All the optical readout components and the piezo-driven stage are mounted on the rotating platform and rotate together during characterization of rotational responses

We used a straightforward microfabrication sequence that relied on single-layer photolithography and deep reactive etching as the main steps. The CVG geometry was a V-shaped tuning fork type with two elliptical features at the end of each tine. The process flow started with photolithographic patterning of CVG shapes on a 100-mm Si wafer that had 1 μm of thermally grown SiO_2_ on both sides. After exposing and developing the photoresist (SPR 220-3.0 MicroChem Inc.), the patterns were etched into the oxide on the front side of the wafer using fluorine-based (CHF_3_/O_2_ or C_4_/F_8_ processing gases) reactive ion etching. Once the oxide was etched through the patterned areas, the wafers were processed using a deep reactive etch (Bosch process). The etch process continued until the etching through the wafer stopped on the SiO_2_ layer present on the backside of the Si wafer. The final step included removal of any residual photoresist with oxygen plasma and removal of SiO_2_ with a solution of hydrofluoric acid. Using the processing sequence outlined earlier, we fabricated a series of V-shaped CVG structures with varying sizes. A wafer with the fabricated CVG structures is shown in Fig. 8a, and Fig. 8b shows a close-up of one of the CVG structures used in the present study. The CVG structures had a length *l* = 8 × 10^−3^ m, a width *w* = 5 × 10^−4^ m, and a thickness *t* = 2.50 × 10^−4^ m.

### CVG characterization

We characterized individual CVG structures using the experimental setup shown in Fig. 8c, d. The overall size of the system we used in our studies was ~0.2 m^3^ and includes all the measurement equipment, the electronics and the rotation table. The size of the gyroscope MEMS device with the readout lasers and detectors was ~10^−6^ m^3^. The CVG structures used were measured as stand-alone devices in an open-loop configuration using a network analyzer and a lock-in amplifier. The individual CVG structures were secured in an aluminum fixture actuated using a miniature piezo-stack connected to a signal generator. The frequency generator provided either a constant drive frequency or a frequency sweep in the range from ~100 Hz to 20 kHz.

Optical readout techniques for MEMS are typically based on reflecting a focused or collimated laser beam from the free end of a cantilever and subsequently measuring the position of the reflected light spot projected onto a segmented photodiode or position-sensitive photodetector. The use of a spatially sensitive photodiode allows the measurement of the position of the reflected laser spot, which, in turn, allows the determination of the cantilever deformation. This particular implementation of the optical readout method was first proposed for use in atomic force microscopy^49^. The dynamic deformations of the CVG tines were detected and quantified using this contactless optical readout method. To measure the motion of both tines, two separate laser beams were focused on the elliptical features at the ends of each tine, as shown in Fig. 8c. The laser photons reflected from each tine were captured by two position-sensitive detectors. This configuration allowed us to measure the motion of each tine separately (nondifferential measurement) and determine the difference in displacement between the two tines (differential measurement).

Nondifferential and differential signals of the oscillating tines were analyzed in the frequency range from 1 to 20 kHz using a lock-in amplifier. Geometrical evaluations of our setup along with calibration allowed us to convert the PSD output signal into a physically meaningful mechanical deflection of each tine. The conversion coefficient for our configuration was 2.5 mV nm^−1^. A digital camera operating at 60 frames per second was used to visualize the motion of CVG tines under stroboscopic illumination. The complete system was mounted on a stepper motor-driven rotational stage that provided consistent rotational rates in the range from 2.78 × 10^−3^ to 278° s^−1^. The temporal responses of the CVG to rotation were recorded without any additional signal processing or filtering.

Spectral analysis of the motion of the CVG tines was carried out to gain insights into the CVG operation, performance, and noise. The resonance frequencies and quality factors of the CVG modes were evaluated by analyzing the output signal from each PSD using either a spectrum analyzer or a lock-in amplifier and were consistent with the eigenfrequency analysis of the CVG tuning fork geometry.

## Supplementary information

Editorial Summary
