# Supplementary material for: Optically read Coriolis vibratory gyroscope based on a silicon tuning fork
Source: Microsyst Nanoeng. 2019 Oct 21;5:47. doi: 10.1038/s41378-019-0087-9 (PMC6803679; doi:10.1038/s41378-019-0087-9)
Supplement: Supplementary file 1 — Editorial Summary [file 41378_2019_87_MOESM1_ESM.docx]

# *Microsystems & Nanoengineering*

**Micro-scale Coriolis gyroscope: all-mechanical**

A Coriolis force vibratory gyroscope that relies entirely on mechanics demonstrates reduced noise. Micro-scale gyroscopes are used in a wide range of devices, and typically rely on mechanical and electrical principles to operate. However, the electrical component of their operation can interfere with the device, causing noise. Now, N.V. Lavrik from Oak Ridge National Laboratory and P.G. Datskos from National Renewable Energy Laboratory demonstrate an all-mechanical device with reduced noise, without the need for signal processing. Key to their design is the use of an optical readout, and a design that takes inspiration from how insects navigate. They achieve a reproducible response to rotational rates as low as 1.8×103 o h-1, and a noise equivalent rate of 0.6 o h-1 for a time of 103 s.

Related article manuscript number: MICRONANO-00795R

Article title: Optically read Coriolis force vibratory gyroscope based on a silicon tuning fork

Corresponding author and affiliation/s: Panos G. Datskos, National Renewable Energy Laboratory, Golden, Colorado, United States

Nickolay V. Lavrik, Oak Ridge National Laboratory, Oak Ridge, TN, United States

**About your Editorial Summary — please read**

**Before approving this Editorial Summary, please carefully check that (1) the summary text lists the correct author(s) and (2) the spelling and order of all author names and affiliations are correct.**

This **Editorial Summary** is based on your manuscript that was recently accepted for publication in *Microsystems & Nanoengineering*. It provides a non-specialist audience with a synopsis of your key research outcomes and conclusions. This value-added service provided by Springer Nature is designed to raise interest in your research across the broader community.

Springer Nature will publish the summary on the journal’s website, and it will be freely available under a under the CC BY licence (Creative Commons Attribution v4.0 International Licence) (see the journal website for details). We encourage you to re-use the summary to bring attention to your research; for example, you can host it on your own website and share it via social-networking platforms. Please attribute the summary to *Microsystems & Nanoengineering* and your article (e.g. by providing a link to your article) and do not make derivatives.

Please note that to maximise the usefulness of these summaries they must follow several stringent guidelines:
-- Spelling, punctuation and style are set according to *Nature* editorial guidelines. As this summary is aimed at non-expert readers, some concepts and technical terms will be simplified.
-- Total length must be no more than 135 words. It is likely that not all points in the paper will be covered.
-- The first sentence must be no more than 280 characters, including spaces, to allow use on microblogging sites.
-- The headline must consist of a brief generic subject identifier followed by a short description. No more than 10 words in total.

Please contact the editorial office ([mems_nano@mail.ie.ac.cn](mailto:mems_nano@mail.ie.ac.cn)) immediately with corrections should you find any factual errors in this Editorial Summary.
